# Isolated Spinous Process Fracture: A Commonly Missed Diagnosis

**DOI:** 10.7759/cureus.99356

**Published:** 2025-12-16

**Authors:** Sagar Maheshwari, Muhammad Munir, Babalola Tayo, Aishwarya Subodh, Zahid Khan

**Affiliations:** 1 Radiology, Barking, Havering and Redbridge University Hospitals NHS Trust, London, GBR; 2 Geriatrics, Queen's Hospital, London, GBR; 3 Geriatrics, Barking, Havering and Redbridge University Hospitals NHS Trust, London, GBR; 4 The William Harvey Research Institute, Queen Mary University of London, London, GBR; 5 Cardiology, University of South Wales, Pontypridd, GBR; 6 Cardiology, University of Buckingham, London, GBR; 7 Cardiology, Barts Heart Centre, London, GBR

**Keywords:** anterior fixation, bilateral upper-lower limb weakness, c5-c6 fracture, care of the elderly, cervical mri, cervical spine fracture, clay-shoveler’s fracture, fall injury, geriatric fall, isolated fracture

## Abstract

Spinous process fractures of the cervical spine are generally considered stable injuries, particularly when they resemble the classic clay-shoveler’s fracture, which typically results from indirect traction forces. However, in certain traumatic settings, these fractures may serve as markers of underlying spinal instability.

We report the case of a 93-year-old woman who sustained a fall following a syncopal episode, striking her head on furniture. On initial evaluation, she was neurologically intact, and computed tomography (CT) of the head and cervical spine revealed no clear fracture or dislocation. Within 24 hours, she developed progressive quadriparesis.

Repeat CT demonstrated a subtle C6 spinous process fracture with posterior facet joint subluxation and anterior displacement of C5 on C6. Magnetic resonance imaging (MRI) revealed a C5-C6 fracture-dislocation with marked anterolisthesis and significant ligamentous injury. The patient underwent spinal traction followed by anterior cervical discectomy, fusion, and plating, resulting in substantial neurological improvement.

This case highlights that even subtle spinous process fractures may indicate serious underlying injury. Unlike isolated clay-shoveler’s fractures, trauma-associated spinous process fractures should prompt further evaluation, particularly with MRI, in patients presenting with delayed or evolving neurological deficits. Early recognition of ligamentous instability is essential to prevent secondary deterioration and optimize clinical outcomes.

## Introduction

Isolated spinous process fractures of the cervical spine are generally regarded as benign injuries [1,2], though they may act as “sentinel” findings in more complex or unstable cervical trauma [3]. Management is typically conservative, involving analgesia, activity modification, and short-term immobilization with a soft cervical collar for 4-6 weeks [4-6]. Surgical intervention is uncommon and usually reserved for cases of persistent pain related to displaced or mobile bony fragments [7,8].

These fractures can result from direct trauma, hyperflexion or hyperextension mechanisms, or repetitive stress, as seen in the classic clay-shoveler’s fracture [4,7]. However, identifying associated unstable ligamentous injuries in the absence of overt fractures or segmental misalignment remains diagnostically challenging [9]. While cervical spine injuries without osseous involvement are relatively uncommon [10,11], facet joint subluxations without fracture may comprise up to 10.65% of cervical spine trauma cases [12], and the incidence of occult ligamentous injury is likely underrecognized [9].

The author reports a case of a C6 spinous process fracture associated with an initially missed underlying ligamentous injury at the same level, later resulting in neurological compromise. This case highlights the importance of a high index of suspicion, comprehensive clinical evaluation, and the judicious use of advanced imaging to detect instability and prevent secondary dislocation or delayed neurological deterioration.

## Case presentation

A 93-year-old woman presented following a fall down a flight of stairs secondary to a cardiac syncopal episode, during which she struck her head against furniture. On admission, she reported pain in the neck, back, and hip regions. She was hemodynamically stable and fully alert (Glasgow Coma Scale score of 15), with no active complaints. Neurological examination revealed no motor or sensory deficits, no signs of myelopathy, and no pathological reflexes.

Initial computed tomography (CT) of the head and cervical spine demonstrated mild degenerative changes, including reduced intervertebral disc height and marginal osteophyte formation, but no definite evidence of fracture or dislocation. She was discharged from the hospital.

Within 24 hours, the patient was readmitted with rapid neurological deterioration characterized by flaccid quadriparesis-complete motor loss (Medical Research Council Grade 0/5) in both lower limbs and reduced strength (2/5) in the upper limbs. An urgent magnetic resonance imaging (MRI) scan of the cervical spine revealed a fracture-dislocation at the C5-6 level, with marked anterolisthesis of C5 over C6, facet joint subluxation, and severe spinal cord compression. Repeat CT confirmed these findings, showing posterior facet joint dislocation/subluxation and anterior translation of the C5 vertebral body on C6. The CT scan from the day of admission had shown only mild degenerative changes without visible fracture or dislocation.

**Figure 1:**
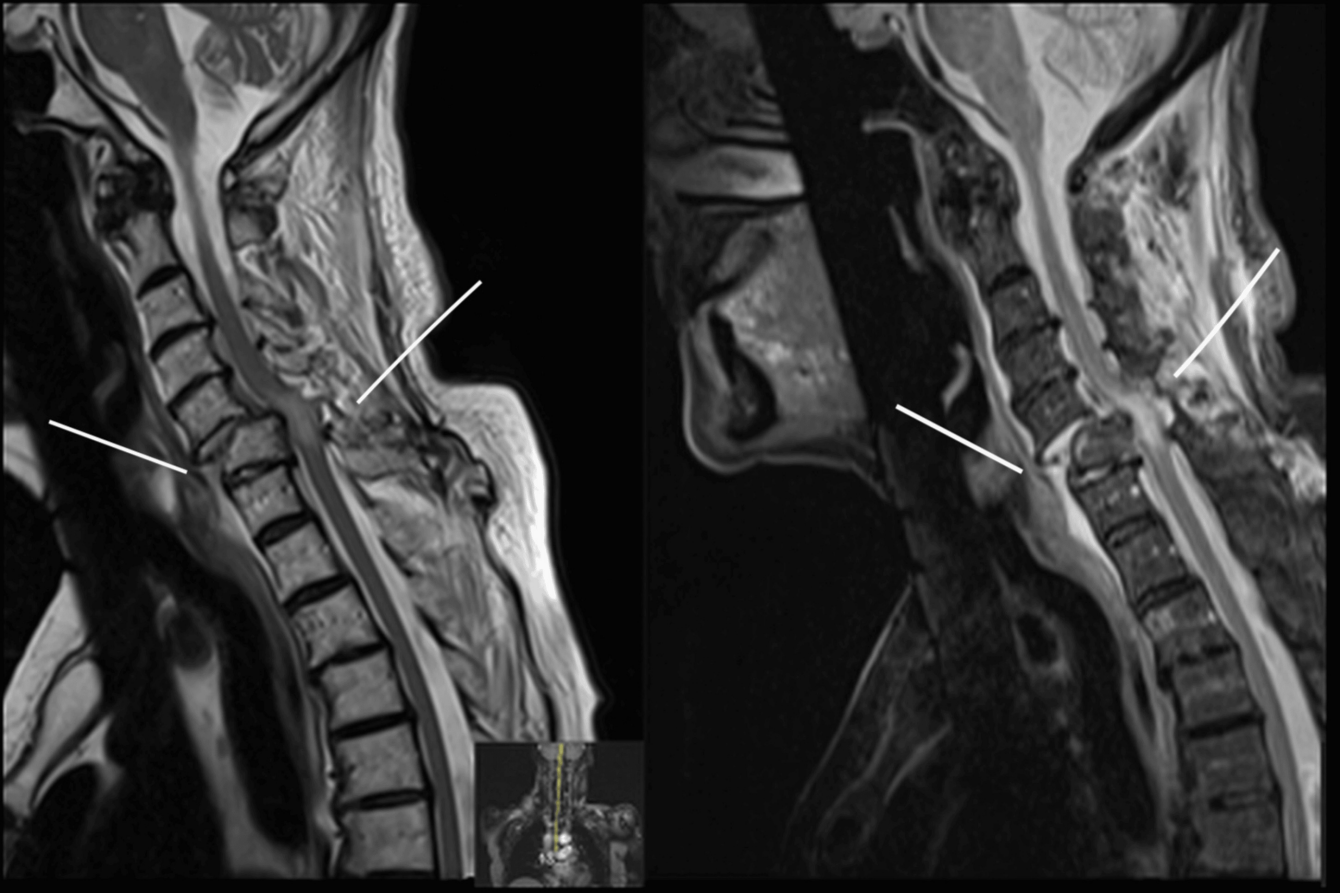
MRI images demonstrating C5-C6 dislocation, with marked anterolisthesis of C5 over C6 and dislocation of the C5-C6 facet joints. There is significant spinal cord compression at this level, with a high STIR signal within the cord. Abnormally high STIR signal is also seen in the prevertebral, paravertebral, and posterior paraspinal soft tissues, consistent with hemorrhage and traumatic injury. STIR: short tau inversion recovery, MRI: magnetic resonance imaging, C5/C6: cervical vertebrae 5/6

MRI confirmed the C5-6 fracture-dislocation with severe cord compression and high short tau inversion recovery (STIR) signal in the spinal cord. An additional high STIR signal in the prevertebral, paravertebral, and posterior paraspinal soft tissues indicated hemorrhage and traumatic soft-tissue injury (Figure 1). 

**Figure 2:**
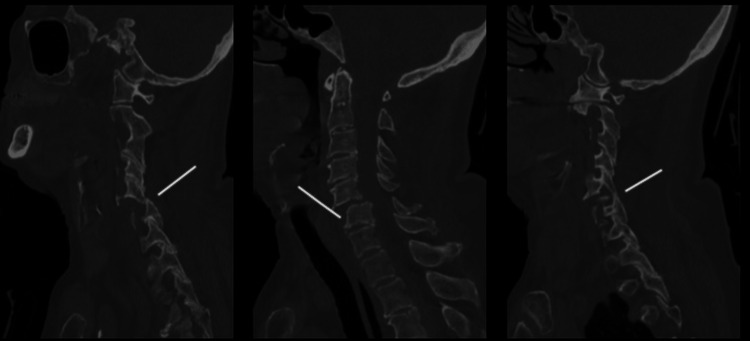
Sagittal CT of the cervical spine showing dislocation of the left facet joints at C5-C6 and perched right facet joint C5-C6, resulting in forward slip of C5 over C6. CT: computed tomography, C5/C6: cervical vertebrae 5/6.

In contrast, post-paralysis CT demonstrated forward slip of C5 on C6 with left-sided facet dislocation, a perched right facet, and small avulsion fragments from the anterior aspect of C6. The posterior border of C6 projected into the neural foramina bilaterally, suggesting possible entrapment of the exiting C6 nerve roots (Figure 2). 

**Figure 3:**
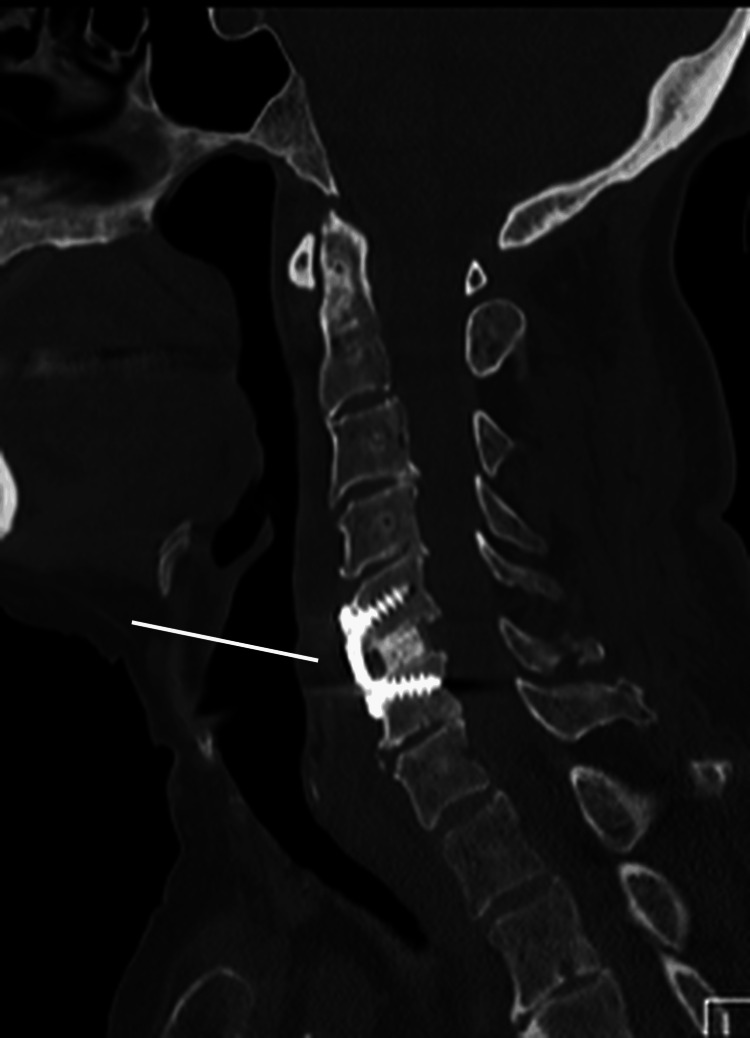
Sagittal CT scan of the cervical spine showing anterior fixation at C5 and C6 levels and position of metalwork and vertebral alignment. CT: computed tomography, C5/C6: cervical vertebrae 5/6.

The patient was initially managed with 7 kg of cervical traction to achieve closed reduction, followed by anterior cervical discectomy and fusion (ACDF) with anterior plating. During surgery, vertebral alignment was restored, and an interbody spacer with a cervical plate and screws was placed at C5-6. Although some widening of the interspinous and bilateral facet joint spaces persisted intraoperatively, the joints remained anatomically aligned without evidence of residual instability. Postoperative imaging confirmed stable construct placement, appropriate sagittal alignment, and intact instrumentation (Figure 3).

The postoperative course was uneventful. Radicular pain resolved completely, and motor function improved to full strength (Medical Research Council Grade 5/5) in all extremities upon discharge.

The patient began early mobilization with physiotherapy focused on cervical stabilization and upper limb strengthening. A follow-up plan was scheduled at three months postoperatively for clinical and radiographic assessment of fusion and neurological recovery. A cervical collar was prescribed for use as needed during the early convalescent period.

## Discussion

Cervical spinous process fractures are uncommon. In the Canadian C-Spine Rule cohort, only 12 such fractures were identified among 8,924 alert trauma patients [1]. Although they can result from direct or indirect trauma, the usual mechanism is avulsion caused by ligamentous stress from trapezius and rhomboid contraction during heavy lifting [6,7]. This classic “clay-shoveler’s fracture” typically involves the C7, C6, or T1 spinous processes and rarely extends into the lamina [2]. While often associated with manual labor, similar injuries occur in athletes [5,6]. Because the posterior ligamentous complex generally remains intact, these fractures are stable and managed conservatively with immobilization and analgesia [2,4-6]. It remains essential to distinguish fractures caused by direct trauma from those due to ligamentous avulsion, as management differs accordingly.

The Canadian C-Spine Rule remains an important tool for assessing alert, stable trauma patients [1]. In the present case, imaging was indicated due to high-risk features, including age over 65, a dangerous mechanism, neck pain, and midline tenderness. Standard protocols begin with three-view radiographs (anteroposterior, lateral, and open-mouth odontoid), followed by CT when radiographs are inconclusive or suspicion persists. Following Barba et al. [13], concurrent head and cervical CT reduces missed injuries. CT is more sensitive and specific than radiographs for detecting bony injury, offering superior diagnostic yield and cost-effectiveness [14-20]. However, CT cannot reliably assess ligamentous disruption or instability [14]. MRI remains the gold standard for evaluating ligamentous and disc injuries [2].

In this case, the initial CT failed to identify a C6 spinous process fracture, which became apparent only on repeat imaging. The fracture, not extending into the lamina, was initially considered stable [1]. Retrospective review, however, revealed subtle but important findings: posterior narrowing of the C5-C6 disc space, discontinuity of the posterior spinolaminar line at C6, and slight ventral laminar displacement. These findings, later confirmed by MRI, reflected posterior annular disruption and posterior ligamentous complex injury. Collectively, they indicated an unstable motion segment that could have been recognized earlier, emphasizing the importance of correlating subtle imaging findings with evolving neurological symptoms.

The delayed dislocation at C6-C7 likely resulted from undetected instability or a spontaneously reduced facet dislocation. One plausible mechanism involves a C6 spinous process fracture with concurrent posterior ligamentous complex and C5-C6 disc disruption, producing progressive instability and eventual unilateral facet dislocation. Alternatively, an initial facet dislocation may have spontaneously reduced, transiently restoring alignment on early imaging. The associated ligamentous and capsular injury likely compromised segmental stability, predisposing the patient to delayed dislocation. Spontaneous reduction of facet dislocations is well documented and may obscure diagnosis on initial assessment [9].

Unilateral facet dislocation is a flexion-distraction injury of the subaxial cervical spine and is typically associated with wide-ranging soft-tissue disruption. MRI often reveals injury to several posterior elements, including the posterior ligamentous complex, ligamentum flavum, facet capsule, and intervertebral disc, while the posterior longitudinal ligament is less consistently affected. Experimental models show that this injury pattern requires failure of both the annulus fibrosus and the ipsilateral ligamentum flavum, highlighting their importance in maintaining stability. The annulus fibrosus, nucleus pulposus, and ligamentum flavum function as major stabilizing structures in this region, and the involvement of multiple stabilizing elements on MRI is strongly associated with mechanical instability, frequently guiding the decision toward surgical stabilization.

This case underscores that even apparently minor cervical spinous process fractures may conceal significant ligamentous or disc injury. The combination of subtle CT findings, progressive neurological symptoms, and confirmatory MRI evidence highlights the importance of a high index of suspicion and appropriate use of advanced imaging. Early recognition of ligamentous instability is crucial to prevent delayed dislocation, neurological decline, and the need for urgent surgical intervention.

## Conclusions

Spinous process fractures should be clearly categorized based on their underlying mechanism-whether caused by direct or indirect trauma, or by ligamentous stress, as in clay-shoveler’s fractures. This distinction is vital for guiding appropriate management strategies. In patients with persistent neck pain, evolving neurological symptoms, or changes in clinical presentation, prompt clinical and radiologic re-evaluation is essential to detect occult instability or delayed dislocation.

Even subtle spinous process fractures can be easily missed on initial imaging, yet they may serve as indicators of significant underlying ligamentous disruption and potential instability. Missing such injuries can lead to delayed diagnosis, secondary dislocation, and neurological deterioration. MRI plays a critical role in these scenarios, as it can reveal soft-tissue and ligamentous injuries not apparent on CT. Early MRI assessment enables timely recognition of instability, appropriate surgical planning when necessary, and prevention of potentially devastating neurological outcomes.
